# Mirage or oasis? Iranian immigrant nurses share their stories of working in overseas healthcare settings: a phenomenological hermeneutic study

**DOI:** 10.3389/fpsyg.2025.1412783

**Published:** 2025-06-13

**Authors:** Milad Rezaiye, Zohreh Vafadar, Mehdi Jafari-Oori, Fakhrudin Faizi

**Affiliations:** ^1^Nursing Care Research Center, Clinical Sciences Institute, Nursing Faculty, Baqiyatallah University of Medical Sciences, Tehran, Iran; ^2^Medical-Surgical Department, School of Nursing, Baqiyatallah University of Medical Sciences, Tehran, Iran

**Keywords:** emigrants and immigrants, nurses, international, health workforce, qualitative research, transcultural nursing

## Abstract

**Background and objective:**

Despite the significant increase in the emigration of Iranian nurses to other countries, there is still a lack of understanding about their experiences and outcomes within healthcare settings abroad. Therefore, this study aimed to narrate the life stories of Iranian immigrant nurses who have practiced nursing in healthcare settings overseas.

**Methods:**

This study utilized a qualitative design and employed the hermeneutic phenomenological approach to collect and analyze data. A total of 12 in-depth and unstructured interviews were conducted with six Iranian immigrant nurses. The participants were selected through purposive and snowball sampling techniques. The interviews were conducted on Skype from March 2023 to January 2024. The collected data was analyzed using the Van Manen method with the assistance of MAXQDA software. The trustworthiness of the study findings was ensured by following the consolidated criteria for reporting qualitative research.

**Results:**

The experiences of Iranian immigrant nurses working in overseas healthcare settings are a complex blend of challenges and opportunities, often resembling mirages and oases. Through data analysis, this study revealed two main themes: “Immigration Mirage” and “Immigration Oasis.” These themes shed light on the numerous challenges faced by Iranian immigrant nurses as they strive to improve their professional identity.

**Conclusion:**

The experiences of Iranian immigrant nurses working abroad involve challenges such as communication barriers, discrimination, and acculturation, as well as opportunities for professional growth and higher remuneration. Their contributions to global healthcare should be recognized and supported, and addressing their specific needs is crucial for enhancing healthcare systems.

## Introduction

The global nursing shortage, projected to reach nine million by 2030, drives migration from countries like Iran, where economic constraints and job dissatisfaction push nurses to seek opportunities in developed nations (Shamsi and Peyravi, 2020; Shirdel et al., 2025; Ahmadi Chenari et al., 2020). Iranian immigrant nurses, defined as those relocated to work abroad, face cultural and linguistic barriers, such as language difficulties and unfamiliar workplace norms, while renegotiating their professional identity—the psychological process of shaping their professional self amid cultural adaptation (Johnson et al., 2012). These challenges highlight the human dimensions of global healthcare migration. This study aims to explore the lived experiences of Iranian immigrant nurses in overseas healthcare settings, focusing on their cultural adaptation and professional identity using hermeneutic phenomenology.

These unique challenges underscore a gap in the literature, as research on immigrant nurses often focuses on migration drivers or quantitative outcomes like job satisfaction (Luma Ghazi Al et al., 2020; Ung et al., 2024), with limited attention to Iranian nurses' subjective experiences (Ahmadi Chenari et al., 2020). Studies highlight cultural and linguistic barriers, discrimination, and acculturation challenges, but rarely use phenomenological approaches to capture lived narratives (Cayaban and Bumalay, 2021). By employing a novel hermeneutic phenomenological approach, this study fills this gap, examining Iranian nurses' cultural adaptation, professional identity, and resilience in overseas settings, advancing psychological research on migration and identity.

This research gap calls for a methodology suited to uncovering nuanced experiences. Hermeneutic phenomenology, rooted in interpretive philosophy (Van Manen, 2023; Gadamer and Linge, 1977), is ideal for exploring the meaning of these nurses' experiences within socio-cultural contexts, offering insights beyond quantitative data. This approach guides our in-depth exploration of their realities.

Grounded in this methodology, we amplify the voices of Iranian nurses in hospitals, clinics, and long-term care facilities in foreign countries selected for significant Iranian migration. Their journeys, marked by cultural dislocation and resilient adaptation, reveal the complexities of global healthcare. This study deepens understanding of migration, identity, and adaptation, informing culturally responsive support for immigrant nurses.

## Methods

### Design and setting

This study adopts a qualitative, hermeneutic phenomenological approach to explore the lived experiences of Iranian immigrant nurses in overseas healthcare settings. Hermeneutic phenomenology, rooted in Wilding and Whiteford's (2005) concept of being-in-the-world and Gadamer's (1975) hermeneutic circle, emphasizes the interpretive co-construction of meaning between researchers and participants (Zahavi and Martiny, 2019). This framework was chosen to uncover the subjective meanings and interpretations Iranian nurses assign to their experiences of cultural adaptation and professional identity in global healthcare contexts, aligning with nursing's focus on human phenomena (Fuster Guillen, 2019). The study design, data collection, and analysis were guided by this philosophy, prioritizing iterative dialogue and contextual sensitivity to reveal the essence of participants' narratives.

### Reflexivity

To enhance credibility, the research team engaged in reflexive practices to address preconceptions and biases. The principal researcher, with a background in Iranian nursing, maintained a reflective journal to document assumptions about migration and cultural challenges, which were discussed in weekly team meetings. These reflections informed interview questions and analysis, ensuring interpretations remained grounded in participants' narratives rather than researchers' prior experiences. Peer debriefing further mitigated bias by challenging initial interpretations, fostering a balanced analysis.

### Participants

The study included six Iranian immigrant nurses practicing in hospitals, clinics, or long-term care facilities in foreign countries. Participants were selected using purposive and snowball sampling to ensure diversity in age, gender, educational level, and nursing experience in Iran and abroad. Eligibility criteria required: (a) at least 2 years of nursing experience in Iran, (b) at least 2 years in an overseas healthcare setting, and (c) fluency in Persian for effective communication. The Corresponding Author purposively selected the first participant, who had 7 years of experience in Iran and Canada. Participants 3, 5, and 6 were recruited via snowball sampling, recommended by initial interviewees.

The depth of insights gathered from in-depth, hour-long interviews, combined with the homogeneity of the participant group's shared Iranian nursing and migration experiences, enabled data saturation after six participants, as no new themes emerged beyond the fifth interview. Saturation was determined through iterative analysis, with recurring themes consistently appearing across narratives by the fifth interview, confirmed by a sixth to ensure completeness. This rigorous process, leveraging the group's shared context and rich narrative data, ensured the sample size adequately captured the complexity of their lived experiences.

### Data collection

Data were collected through in-depth, individual interviews conducted via Skype, allowing flexibility and accessibility. After building trust through two pre-interview meetings to explain the study's purpose and address concerns, informed consent was obtained via email, with confidentiality assured. Interviews began in an unstructured format to encourage spontaneous narratives, transitioning to a semi-structured format after initial interviews to explore emerging themes (e.g., professional identity struggles). This shift enhanced data depth by focusing on specific experiences while preserving phenomenological flexibility.

Interview questions were tailored to elicit lived experiences, including: “How has your Persian identity shaped your nursing practice abroad?” “What challenges arise when navigating cultural differences in patient care?” and “How do others' perceptions of you as an immigrant nurse affect your professional identity?” Probing questions, such as “Can you describe a moment when cultural differences felt overwhelming?” or “What emotions surfaced when facing workplace marginalization?” deepened responses. Interviews, lasting 60–90 min, were audio-recorded with consent and transcribed verbatim, continuing until saturation was reached after six participants.

Steps of conducting interviews:

1. Participant recruitment: collaborating with Iran's Ministry of Health, we accessed a list of Iranian immigrant nurses, using purposive sampling to select eligible participants and snowball sampling to expand recruitment.
2. Pre-interview meetings: two virtual meetings-built rapport, clarify the study's aims, and address participant concerns.
3. Interview preparation: initial unstructured interviews allowed free expression, followed by a semi-structured question guide to probe emergent themes.
4. Conducting interviews: Skype interviews balanced spontaneity and guided exploration, using tailored questions and probes.
5. Recording and transcribing: audio recordings were transcribed verbatim, with transcripts validated by participants.

### Data analysis

The collected data was then analyzed to create initial concepts and themes, which then guided the subsequent semi-structured interviews. The process of data analysis followed a rigorous and systematic approach, using Van Manen's hermeneutic phenomenological method (Van Manen, 2023; Gerchow et al., 2021). A depiction of the coding process can be found in Table 1.

**Table 1:** The process of emerging themes.

**Theme**	**Sub-themes**	**Sub-sub-themes**	**Meaning units**
Immigration oasis	Professional identity	Professional attitude	Having professional responsibility, nursing beyond borders
		Superiority complex	Sense of excellence toward the profession, satisfaction with clinical practices
		Moral vigilance	Sensitivity to meeting the physical needs of patients, sensitivity to meeting the psychological needs of patients
	Organizational norms	Environmental justice	Ensuring a workplace free from racism, ensuring equity among diverse medical personnel
		Labor standards	Ensuring nurses have access to adequate resources and facilities, ensuring compliance with nursing work environment standards, and fulfilling specific nursing duties in line with job descriptions
		Systematic professionalization	Enhancing the nursing laddering system, enhancing specialized nursing care

Van Manen's approach consists of six steps:

1. Turning to the nature of lived experience: this first step involves focusing on the participants' lived experiences to understand the phenomenon under investigation.
2. Investigating experience as we live it: here, researchers reflect on their own experiences in relation to the topic, thus gaining insights that can inform the analysis.
3. Reflecting on the essential themes: this step requires identifying and articulating the themes that emerge from the data, capturing the essence of the lived experiences shared by participants.
4. Describing the phenomenon through the themes: in this stage, the researcher crafts a narrative that encapsulates the themes identified, providing a rich and nuanced description of the phenomenon.
5. Maintaining a reflective attitude: throughout the analysis, it is crucial to remain reflective and open to new insights, allowing for a deeper understanding of the participants' experiences.
6. Writing the reflective narrative: the final step involves synthesizing the findings into a coherent narrative that conveys the essence of the participants' experiences, highlighting the themes identified in the previous steps.

By employing Van Manen's approach, we were able to rigorously analyze the narratives of Iranian immigrant nurses and uncover the complexities of their experiences in overseas healthcare settings.

### Rigor

The study findings were upheld with rigor by adhering to the consolidated criteria for qualitative reporting research (De Witt and Ploeg, 2006; Booth et al., 2014). To ensure the authenticity of the findings, the research team first identified and addressed any biases. For credibility, the team engaged extensively with participants and the topic, carefully reviewing each interview multiple times. Additionally, the findings were shared with participants for validation through member checks. Regular contact via phone or Skype was maintained to ensure a clear understanding of participant statements, clarify experiences, and address any ambiguities to establish a shared understanding of experiences. Peer debriefing sessions were conducted to review and discuss findings, facilitating consensus among the research team. Open-ended interviews were conducted to enhance confirmability. To ensure dependability, transparent reporting was achieved through audio recording and transcription of interviews. For increased transferability, detailed step-by-step descriptions of the research process and characteristics of the studied population were provided to aid other researchers in replicating the study.

### Ethical considerations

This study received approval from Baqiyatallah University of Medical Sciences (IR: BMSU.REC.1401.114). Before conducting the interviews, participants were informed about the recording process and given informed consent via email. Participants were assured that they had the right to withdraw from the study at any time without facing any consequences. To protect confidentiality, participants' identities were safeguarded using unique codes, such as numerical identifiers.

### Findings

#### Participant characteristics

Participants had an average age of 38.16 years (SD = 4.62), 8.66 years of nursing experience in Iran (SD = 2.50), and 4.41 years in overseas healthcare settings (SD = 2.87). Three held a BSc in Nursing, two an MSc in Intensive Care Nursing, and one an MSc in Pediatric Nursing (see Table 2). Six Iranian immigrant nurses from hospitals, clinics, or long-term care facilities in Canada, Germany, the UAE, and Australia were interviewed, achieving data saturation. Two additional nurses were interviewed to confirm replicability, reinforcing the robustness of the findings. Data analysis yielded two main themes: “Immigration Mirage” and “Immigration Oasis” (see Table 3).

**Table 2:** Sociodemographic characteristics of the participants (*N* = 6).

**Participants**	**Age (years)**	**Gender**	**Educational level**	**Nursing experience in Iran (Years)**	**Nursing experience in overseas country (Years)**	**Migrated country**	**Interview duration (min)**
P1	44	Female	BSc	7	7	Canada	85
P2	39	Male	BSc	10	2.5	Germany	78
P3	37	Female	BSc	12	3	UAE	76
P4	42	Female	MSc in Pediatric Nursing	10	9	Canada	79
P5	36	Female	MSc in Intensive Care Nursing	8	2	Germany	77
P6	31	Female	MSc in Intensive Care Nursing	5	3	Australia	72

**Table 3:** Extracted themes, sub-themes, and sub-sub-themes.

**Theme**	**Sub-themes**	**Sub-sub-themes**
Immigration oasis	Professional identity	Professional attitude
		Professional independence
		Professional behavior
		Superiority complex
		Moral vigilance
	Organizational norms	Environmental justice
		Legalities
		Labor standards
		Healthcare quality standards
		Systematic professionalization
	Wellbeing	Social valuation
		Psychological support
		Positive cooperation
		Safety and Security
		Sociability
Immigration mirage	Gaps between individual and organizational goals	Non-spiritual nursing
		Bias perception
		Being neglected belief
		Strict responsible behavior
		Feeling incompetent
	Cultural-linguistic duality	Feeling pathetic
		Unethical Behavior in the Workplace
		Feeling ambivalence
		Acculturation
		Interactional barriers
	Feeling Stuck in Adjusting to Change	Hard career adjustment
		Isolation
		Demotivation
		Guilt
		Repatriation rumination

#### Main themes

Using van Manen's hermeneutic phenomenological method, we iteratively analyzed participants' narratives to uncover the essence of their lived experiences as Iranian immigrant nurses. Initial coding identified meaning units (e.g., expressions of cultural struggle or professional pride), which were clustered into sub-sub-themes (e.g., bias perception, moral vigilance) based on shared meanings. These were grouped into sub-themes (e.g., Cultural-Linguistic Duality, Professional Identity) through reflective dialogue, synthesizing into two main themes: “Immigration Mirage,” capturing barriers to adaptation (e.g., cultural disconnection, demotivation), and “Immigration Oasis,” reflecting enablers of professional identity and wellbeing (e.g., autonomy, support). This structure, detailed in Table 1, contrasts the challenges and opportunities shaping nurses' professional identities in overseas settings.

#### Immigration oasis

Iranian immigrant nurses described how immigration enhanced their professional identity by adapting to overseas healthcare norms, improving care quality and job satisfaction. Respectful treatment and workplace support fostered personal and social wellbeing, reinforcing their commitment to nursing. Three sub-themes emerged under Immigration Oasis: “Professional Identity,” “Organizational Norms,” and “WellBeing”.

### Professional identity

#### Professional attitude

Organizational mandates for full patient responsibility cultivated a deep sense of duty, transcending cultural, linguistic, and gender boundaries. This holistic approach fostered a professional attitude, reflecting nurses' commitment to patient care.

*When providing nursing care, I prioritize the wellbeing of my patients above all, regardless of their gender, language, culture, or nationality. This approach embodies the true essence of nursing, as we wholeheartedly dedicate ourselves to their care and comfort*. (P5)

##### Professional independence

Significant authority in patient care enabled nurses to design and monitor care plans autonomously, enhancing intervention effectiveness. Assigning dedicated nurses from admission to discharge ensured continuity, empowering nurses to track progress and adjust treatments independently.

*One remarkable advantage is that once a patient is admitted, they remain under your care until the day of discharge, regardless of the shift you work. This continuity ensures that the patient's care remains consistent. This allows you to implement and monitor the care plan you have set for the patient, track their progress, such as the healing of a bed sore, and ensure they receive consistent treatment*. (P2)

##### Professional behavior

Constant bedside presence fostered professional behaviors, enabling nurses to provide specialized training on medications, self-care, and equipment use. This engagement enhanced care quality and personal fulfillment.

*I find the experience quite fulfilling. It's not just about ticking off tasks and clocking out. As a caregiver, you are fully present with your patient, guiding them through each moment and shift. You educate them on medications, and available resources, and assist with tasks or teach them self-care. This level of engagement is truly rewarding, in my opinion*. (P3)

##### Superiority complex

Mastery of clinical skills and patient care success instilled a sense of professional superiority, elevating nurses' self-worth and dedication.

*It is undeniable that I hold myself in high regard. I find purpose in being of service to others, my community, my family, and myself*. (P1)

##### Moral vigilance

Sensitivity to patients' physical and psychological needs defined nurses' practice. Compassion, empathy, and respect fostered empathetic connections, elevating care standards.

*As a caregiver, I understand the immense responsibility that comes with providing care to a patient who relies entirely on me. It is crucial for me to be at my best in order to attend to the patient's every need. From ensuring their comfort to assisting with personal hygiene, I must approach each task with a sense of freshness and energy. By dedicating time and effort to the patient, I am not only helping them physically but also providing them with the essential emotional support they require*. (P4)

#### Organizational norms

##### Environmental justice

Nurses valued discrimination-free workplaces where anti-racist policies and shared spaces with doctors promoted fairness and collaboration, reducing hierarchical distinctions.

*No tolerance for racism ensures a fair workplace. I feel valued knowing my contributions are judged on merit, not background*. (P1)

##### Legalities

Defined treatment protocols empowered nurses to execute legal responsibilities confidently, ensuring compliance and professional satisfaction.

*At our facility, we adhere to a comprehensive set of protocols that cover every aspect of patient care. From adjusting heparin to managing potassium levels, each procedure is clearly outlined in a protocol. These guidelines ensure that our work is not only efficient but also compliant with legal standards*. (P4)

##### Labor standards

Appropriate salaries, consistent schedules, and clear job descriptions enhanced job contentment by focusing on core nursing duties.

*As a nurse, I appreciate the clear guidelines that have been set for me to follow. Adhering to these principles ensures that I stay within my scope of practice. I am committed to upholding these standards and providing care within the boundaries they define*. (P5)

##### Healthcare quality standards

Non-discriminatory care, ample equipment, and electronic reporting elevated care quality, enabling tailored patient services.

*There is an abundance of equipment available that enhances the patient unique care experience, making it truly enjoyable to interact with and assist patients. Every individual can receive tailored services based on their unique needs. Also, our nursing reports are meticulously maintained in an electronic format using a standardized template to ensure accuracy and accountability for all recorded information*. (P3)

##### Systematic professionalization of nursing

Education-based laddering and specialized training fostered professional growth and high-quality care delivery.

*Nursing at our facility is characterized by a high level of professionalism. Our nurses are divided into different groups based on their expertise and responsibilities. We have nurses who provide essential care, LPNs who function as assistant nurses, and nursing experts with advanced skills in patient care. Additionally, when patients require specialized care or consultation, we collaborate with nurse practitioners who possess extensive knowledge and experience*. (P1)

#### Wellbeing

##### Social valuation

Respectful treatment and union support enhanced nurses' sense of social status and honor.

*During my month-long absence from work following surgery, the nursing union maintained regular contact with me, offering unwavering support and assistance until I was fully prepared to resume my duties as a nurse. Their continuous help and encouragement were invaluable during my recovery period*. (P4)

##### Psychological support

A welcoming workplace atmosphere promoted wellbeing and productivity, aiding adjustment.

*In the hospital where I work as a nurse, the staff consistently demonstrates a friendly and supportive attitude. The camaraderie among doctors and nurses has greatly benefited me in my role*. (P2)

##### Positive cooperation

Collaborative teamwork, likened to synchronized bodily systems, fostered gratification and care quality.

*In our team, each member plays a vital role, much like different parts of bodily systems. The nurse, nurse's assistant, and doctor each have their specific duties, working together seamlessly. Our collaborative efforts mirror the synergy of interconnected body parts. Our strong working relationship further enhances our effectiveness and the quality of care we provide*. (P6)

##### Safety and security

Strict security measures ensured a safe work environment, alleviating concerns about violence.

*Here at our facility, we maintain strict security measures to ensure safety. Our dedicated security guard oversees all activities diligently. Just recently, during my night shift, a patient with a mental disorder became agitated and overturned my desk. I promptly contacted the security team, who promptly intervened and safely escorted the patient back to their room. Following proper protocol, I administered necessary medication, leading to the patient's eventual calming down*. (P1)

##### Sociability

Cultural competence training and resources supported nurses' societal integration.

*In addition to the cultural orientation classes they provide, the hospital generously offers us expensive social books for free to help us assimilate into the society. This support is invaluable in helping us adapt to the new culture*. (P2)

#### Immigration mirage

Iranian immigrant nurses faced significant barriers to adaptation, including behavioral, emotional, and cognitive challenges that hindered performance and prompted some to consider repatriation. These struggles, rooted in cultural and professional disconnection, contrasted with the enabling experiences of “Immigration Oasis.” Three sub-themes emerged under Immigration Mirage: “Gaps between Individual and Organizational Goals,” “Cultural-Linguistic Duality,” and “Feeling Stuck in Adjusting to Change.”

#### Gaps between individual and organizational goals

##### Non-spiritual nursing

Nurses experienced a loss of nursing's spiritual significance abroad, unlike its revered status in Iran, creating a profound professional disconnect.

*Here nursing is often seen as just another job without any special recognition. In contrast, in Iran, nursing is revered as a spiritual and sacred profession*. (P4)

##### Bias perception

Hidden and overt biases persisted despite anti-discrimination laws, causing frustration and alienation.

*In the work environment, there seems to be a lack of clarity in their actions, indicating the presence of underlying biases*. (P4)

##### Being neglected belief

Nurses felt invisible and unappreciated, diminishing workplace enjoyment despite increased efforts.

*I still haven't received the same level of encouragement in my current environment as I did back in Iran, despite doubling my efforts. Consequently, I find it challenging to derive much enjoyment from my current situation*. (P6)

##### Strict responsible behavior

The heavy burden of patient responsibility, while fostering duty, was overwhelming, contributing to mental and physical exhaustion.

*Taking on full responsibility for a patient can feel overwhelming to me. The weight of knowing that their care rests solely on my shoulders can be daunting. I find myself shouldering numerous responsibilities, which can feel quite heavy*. (P4)

##### Feeling incompetent

Work pressures and unmet expectations led to feelings of incompetence, frustrating nurses' ability to apply their skills.

*I often feel useless in this environment as I am unable to utilize my known skills effectively*. (P6)

#### Cultural-linguistic duality

##### Feeling pathetic

Colleagues' pitying attitudes undermined nurses' confidence, reinforcing feelings of being undervalued.

*Instead of offering genuine support, they often look at me with pity and dismiss my abilities by saying, “You don't know, you can't do it, and we will cover for you.” This lack of understanding and condescending attitude leaves me feeling disheartened and undervalued*. (P6)

##### Unethical behavior in the workplace

Gossip and false reporting by colleagues created a disillusioning work environment, clashing with nurses' expectations.

*The first time I encountered gossip in the workplace, I was taken aback. I found it surprising that such behavior could occur among colleagues in a developed country. It left me feeling quite upset*. (P6)

##### Feeling ambivalence

Nurses felt conflicted about basic care tasks (e.g., hygiene), valuing patient attention but finding them unpleasant.

*Cleaning the patient's feces is not pleasant for me at all, although I really like it because it makes you pay more attention to your patient*. (P5)

##### Acculturation

Navigating cultural misconceptions (e.g., as Muslim women) was a persistent challenge, requiring active efforts to integrate and educate others, often without reciprocal understanding.

*Some may avoid greeting or making eye contact with me as a Muslim woman. I actively work to dispel these misconceptions, educating and integrating myself into the cultural norms of this society*. (P3)

##### Interactional barriers

Communication challenges with patients and colleagues caused stress and misunderstandings, hindering care quality and reinforcing the Mirage of adaptation struggles.

*When it comes to nursing tasks that require explaining procedures to patients, showing empathy, or educating them, I often face challenges that lead to significant pressure and stress*. (P5)

#### Feeling stuck in adjusting to change

##### Hard career adjustment

Unfamiliar protocols and administrative complexities impeded integration into new healthcare systems.

*Adapting to the treatment system here has proven to be quite challenging for me. I was accustomed to relying on the doctor's opinion in my previous treatment experiences, which made it difficult for me to transition to a system that required me to make decisions based on existing treatment protocols*. (P1)

##### Isolation

Limited social connections with colleagues fostered isolation, reducing workplace joy and care quality.

*I struggle to have fun in my workplace but I find it difficult to join in my colleagues' laughter. in Iran, I found joy in connecting with colleagues, sharing memories, exchanging news, and good-naturedly teasing each other. However, here, while these interactions occur, I often feel like I'm on the outside looking in. It's as if I'm the odd one out, experiencing a sense of disconnect from those around me*. (P6)

##### Demotivation

Multiple obstacles, including harsh work conditions, sapped nurses' enthusiasm, deepening their sense of disconnection.

*Each day feels heavier, with constant barriers draining my motivation to engage fully in my role*. (P5)

##### Guilt

Challenges in meeting patient needs fueled guilt and vulnerability, undermining self-advocacy.

*Feeling guilty about potentially not being able to provide optimal care for a patient or showcase my abilities is a concern*. (P5)

##### Repatriation rumination

Frequent thoughts of returning to Iran, driven by adaptation struggles, reflected nurses' longing for familiar professional and cultural contexts.

*Sometimes I dream of nursing back home, where my work felt meaningful and understood*. (P6)

## Discussion

This study aimed to explore the lived experiences of Iranian immigrant nurses in overseas healthcare settings, focusing on their professional identity formation and adaptation challenges. Using a hermeneutic phenomenological approach, we identified two primary themes: “Immigration Mirage,” capturing barriers such as cultural-linguistic duality and organizational misalignment, and “Immigration Oasis,” reflecting enablers like professional autonomy and supportive norms. These findings illuminate the dual nature of nurses' experiences, revealing both persistent struggles and opportunities for growth that shape their professional identities.

### Immigration mirage

The “Immigration Mirage” theme underscores the disconnect between Iranian immigrant nurses' personal aspirations and organizational goals, eroding their professional identity. In Iran, nursing is imbued with spiritual significance, yet abroad, nurses often perceive it as a utilitarian role, lacking sacred value. This aligns with Shirzad et al. (2020), who highlighted Iranians' spiritual inclinations and interest in integrating spirituality into clinical practice (Shirzad et al., 2020). Murgia et al. (2020) notes that multicultural societies interpret spirituality fluidly, often prioritizing materialistic values, which creates ambiguity for nurses accustomed to a spiritually rich practice (Murgia et al., 2020). This cultural dissonance, compounded by other challenges, fosters a sense of professional alienation.

Discrimination and neglect further intensify this disconnect. Antón-Solanas et al. (2022) found that immigrant nurses face biases based on culture, race, and language, hindering workplace contributions (Antón-Solanas et al., 2022). This discrimination hinders their ability to fully contribute to their workplaces and achieve their personal and professional goals (Pressley et al., 2022). Many Iranian immigrant nurses report feelings of neglect, which contributes to a growing misalignment between their aspirations and organizational objectives, leading to a decline in motivation. Hamed et al. emphasize that this sense of being deprioritized is a common challenge among immigrant nurses, creating significant barriers to their success (Hamed et al., 2020). These experiences collectively undermine professional identity by limiting nurses' sense of belonging and value.

Additionally, Iranian immigrant nurses often find it difficult to meet the strict responsibilities of their roles, which can lead to feelings of inadequacy. Farokhzadian et al. stress the need for practical strategies to ensure patient safety in developed countries, where the diligent implementation of nursing practices has significantly reduced patient harm compared to developing nations (Farokhzadian et al., 2018). This stark contrast can make nursing in developed countries feel particularly daunting for Iranian immigrant nurses.

Cultural-linguistic duality presents another significant challenge for these nurses, impacting their caregiving performance and overall wellbeing. Language barriers can lead to misunderstandings and frustration, manifesting as pitying looks from colleagues. Numerous studies have identified culture and language as major challenges for immigrant nurses (Gerchow et al., 2021; Schouten et al., 2020; Balante et al., 2021). Pung et al. highlight the critical role language plays in building relationships and shaping social identities, noting that limited proficiency can impede effective communication (Pung and Goh, 2017). Furthermore, Iranian immigrant nurses often encounter cultural misunderstandings and unethical behaviors from colleagues and patients, leading to ambivalence in their caregiving. Rodríguez et al. argue that the dual challenge of acclimating to a new culture and professional environment diminishes the sense of empowerment among immigrant nurses (Rodríguez et al., 2014).

Despite these challenges, Iranian immigrant nurses also experience the “Immigration Oasis” phenomenon, where their practice in overseas care settings plays a vital role in shaping their professional identity. Nursing instills a strong dedication that transcends geographical boundaries, providing them with significant responsibilities. Their ability to deliver effective care independently fosters a sense of professional autonomy. Through their vigilance at patients' bedsides and their focus on education and specialized care, they demonstrate exemplary professional conduct. Their moral sensitivity is evident in their attentiveness to both the physical and psychological needs of their patients, leading to increased satisfaction and confidence in their clinical performance. This aligns with findings from Seo and Kim, who emphasized the importance of professional identity and commitment to patient care among Korean nurses in the United States (Seo and Kim, 2017). However, contrasting findings from Deegan and Simkin reveal that immigrant nurses in Australia who struggle with English proficiency often feel disempowered and face barriers in reclaiming their professional identity (Deegan and Simkin, 2010). It is vital to recognize that the erosion of professional identity may be linked more to communication challenges than to the care environments themselves.

### Immigration oasis

The “Immigration Oasis” concept is also characterized by establishing supportive organizational norms. Iranian immigrant nurses often find their workplaces to be equitable environments, where distinctions between doctors and nurses are minimized. They appreciate the systematic approach and legal adherence in their work settings, which bolsters their professional performance. Additionally, they value appropriate facilities and competitive salaries. Their commitment to quality care is reflected in their meticulous work, such as thorough nursing reports and the use of advanced equipment. The presence of a structured career advancement system based on education and specialization further enhances the professionalization of nursing in developed countries. Mengstie's research underscores the importance of organizational norms, particularly structural justice, in healthcare settings, emphasizing the need for fair payment and equitable treatment to retain immigrant nurses (Mengstie, 2020). Establishing favorable working conditions that promote fairness and justice is crucial for enhancing immigrant nurses' retention.

Moreover, Iranian immigrant nurses experience positive wellbeing in overseas settings, where they are treated with dignity and respect. The close relationships formed in the workplace, coupled with emotional support, contribute significantly to their overall sense of peace. Strong camaraderie among medical staff enhances collaboration, while initiatives aimed at improving the socialization of immigrant nurses foster a sense of integration within established norms. Dahl et al. found that immigrant nurses express optimism about enhancing their professional skills and navigating the legal frameworks within their respective healthcare systems (Dahl et al., 2017). These findings align with Wesołowska et al., who noted that empathy and effective communication are vital for supporting the wellbeing of immigrant nurses, reducing stress and anxiety (Wesołowska et al., 2018). Overall, the literature consistently highlights that fostering an empathetic work environment and providing emotional support can significantly enhance the wellbeing of immigrant nurses (Pung and Goh, 2017; Chok et al., 2018; Liem et al., 2021).

## Study strengths

This study's hermeneutic phenomenological approach, guided by van Manen's method, rigorously captured the nuanced lived experiences of Iranian immigrant nurses, a relatively understudied population. By focusing on this group, the study addresses a gap in the literature, offering insights into the unique cultural and spiritual dimensions of their adaptation. The iterative analysis, supported by member checks and peer debriefing, ensured credibility and depth. These findings enrich global healthcare workforce dynamics by highlighting the interplay of cultural competence, professional identity, and organizational support, informing policies to enhance immigrant nurse retention and wellbeing in diverse settings.

## Limitation

Recruiting Iranian immigrant nurses was challenging due to limited access and trust issues, with 11 potential participants unable to commit time. Collaboration with Iran's Ministry of Health and two pre-interview meetings mitigated this by building rapport and emphasizing the study's policy relevance. While sufficient for phenomenological depth, the small sample size (six participants) limits transferability, though saturation was achieved. Skype interviews, while accessible, occasionally faced technical disruptions (e.g., audio delays) and limited non-verbal cues, potentially reducing data richness. These were addressed by verbatim transcription and participant transcript validation. The focus on clinical experiences excluded family-related challenges, warranting future research. The scarcity of male participants, some reluctant to share emotions, may have skewed findings toward female perspectives. Potential researcher biases, such as cultural assumptions from the principal researcher's Iranian nursing background, were mitigated through reflective journaling and team discussions. Future studies should explore male nurses' experiences and family dynamics.

## Conclusion

This study explores the experiences of Iranian immigrant nurses who work in healthcare settings abroad. It provides insight into the challenges they face and the opportunities they encounter.

Working in a foreign country's healthcare system can seem appealing, offering better prospects and a brighter future. However, the reality is often more complex. Iranian immigrant nurses often struggle with communication barriers, cultural differences, and the need to adapt to new healthcare protocols and practices. As a result, they may feel isolated and frustrated, questioning whether returning to their home country is the best option.

On the other hand, working in overseas healthcare settings can sometimes be a positive experience for Iranian immigrant nurses. They have opportunities for professional advancement, higher compensation, and the chance to make a meaningful impact in their field. Exposure to diverse patient populations and advanced medical technologies can enhance their skills and knowledge, ultimately enriching their professional identity.

The experiences of Iranian immigrant nurses in overseas healthcare settings are multifaceted, and it is important to recognize the valuable contributions they make to the global healthcare workforce. Their diverse perspectives, cultural competencies, and unwavering dedication to patient care enrich healthcare settings worldwide.

Ultimately, the experiences of Iranian immigrant nurses in overseas healthcare settings involve both challenges and opportunities, offering both mirage and oasis. By understanding and addressing the unique needs of these nurses, healthcare systems can fully utilize their contributions while ensuring their wellbeing and professional fulfillment.

## Data Availability

The datasets presented in this study can be found in online repositories. The names of the repository/repositories and accession number(s) can be found in the article/supplementary material.
